# *Eragrostis curvula*, a Model Species for Diplosporous Apomixis

**DOI:** 10.3390/plants10091818

**Published:** 2021-08-31

**Authors:** Jose Carballo, Diego Zappacosta, Juan Pablo Selva, Mario Caccamo, Viviana Echenique

**Affiliations:** 1 Centro de Recursos Naturales Renovables de la Zona Semiárida (CERZOS–CCT–CONICET Bahía Blanca), Camino de la Carrindanga km 7, Bahía Blanca 8000, Argentina; jcarballo@cerzos-conicet.gob.ar (J.C.); jpselva@criba.edu.ar (J.P.S.); echeniq@cerzos-conicet.gob.ar (V.E.); 2 Departamento de Agronomía, Universidad Nacional del Sur (UNS), San Andrés 800, Bahía Blanca 8000, Argentina; 3 Departamento de Biología, Bioquímica y Farmacia, Universidad Nacional del Sur (UNS), San Juan 670, Bahía Blanca 8000, Argentina; 4 NIAB, 93 Lawrence Weaver Road, Cambridge CB3 0LE, UK; Mario.Caccamo@niab.com

**Keywords:** cytoembryology, ovule development, reproduction

## Abstract

*Eragrostis curvula* (Schrad.) Ness is a grass with a particular apomictic embryo sac development called Eragrostis type. Apomixis is a type of asexual reproduction that produces seeds without fertilization in which the resulting progeny is genetically identical to the mother plant and with the potential to fix the hybrid vigour from more than one generation, among other advantages. The absence of meiosis and the occurrence of only two rounds of mitosis instead of three during embryo sac development make this model unique and suitable to be transferred to economically important crops. Throughout this review, we highlight the advances in the knowledge of apomixis in *E. curvula* using different techniques such as cytoembryology, DNA methylation analyses, small-RNA-seq, RNA-seq, genome assembly, and genotyping by sequencing. The main bulk of evidence points out that apomixis is inherited as a single Mendelian factor, and it is regulated by genetic and epigenetic mechanisms controlled by a complex network. With all this information, we propose a model of the mechanisms involved in diplosporous apomixis in this grass. All the genetic and epigenetic resources generated in *E. curvula* to study the reproductive mode changed its status from an orphan to a well-characterised species.

## 1. Introduction

*Eragrostis curvula* (Schrad.) Ness is a C4 species member of the Poaceae family, Chloridoideae subfamily. This grass belongs to the monophyletic Eragrostideae tribe; however, Eragrostis as well as other genera in the Chloridoideae subfamily is polyphyletic, since the species of this genus are in different clades associated with other genera like Ectrosia and *Uniola* [1]. The Eragrostideae tribe contains three sub-tribes, Cotteinae, Eragrostidinae, and Uniolinae. Eragrostis is the largest genus of the Chloridoideae subfamily, containing approximately 400 species [2,3]. *Eragrostis tef,* another important species of the genus, is a staple food in Ethiopia and produces 4.5 million tons over 2.9 million hectares [4]. *E. curvula* is used as forage grass for semi-arid regions in the USA, Australia, and Argentina among others [5].

The *E. curvula’s* origin centre is located in southern Africa along with other Chloridoideae species [1,6]. This grass was subsequently distributed in all continents [7] and in some places such as North America and Australia has become an invasive weed, and its biology is investigated in order to avoid its expansion [5,8,9]. *E. curvula* was considered for a long time as a complex since the morphological characteristics of the species are highly variable [10]. This complex was composed of *E. curvula*, *E. lehmaniana* and *E. chloromelas*. However, the molecular characterisation of the species could resolve this issue, and now the species is well circumscribed [1,3,11]. The germplasm available at the moment at the USDA seedbank comprises 550 accessions and our collection at CERZOS-CONICET (https://cerzos.conicet.gov.ar/, accessed on 21 July 2021) contains 70 accessions. Although *E. curvula* has been studied in relation to its drought stress resistance [12,13,14,15,16], forage quality [17,18,19,20,21], and soil erosion [22,23], it also offers a peculiar reproductive mode, apomixis.

The first time that *E. curvula* was reported as apomictic was in 1958 in a survey of a wide range of grasses in which, through a cytoembryological examination, this species was classified as apomictic [24]. The second report was in 1963 [25], and after that, in the 1970s, apomixis in *E. curvula* was described in detail [26,27,28]. These findings were the foundation for further investigations of the reproductive mode of *E. curvula*, and several studies have been published since then using different approaches, such as tissue culture [29], molecular markers [30], development of near-isogenic genotypes with different ploidy levels by in vitro culture [31,32], transcriptomics [33,34,35], genotyping [36,37], genomics [38,39], and epigenomics [40,41,42,43,44,45,46].

Apomixis is a type of asexual reproduction that produces seeds without fertilization in which the progeny is genetically identical to the mother plant. A plant is considered apomictic when at least one seed is produced by apomixis, even though facultative genotypes coexist with sexual and apomictic embryo sacs [47]. Apomictic development includes three main components, which in the last instance would produce an embryo from a non-reduced cell: (i) apomeiosis, i.e., circumvention of meiosis; (ii) parthenogenesis, i.e., embryogenesis without egg fertilization; and (iii) autonomous endosperm formation or pseudogamy, i.e., fertilization of the polar nucleus to form endosperm without fertilization of the egg cell. Apomixis is actually classified as sporophytic (or somatic embryogenesis) and gametophytic [48]. In sporophytic apomixis, the embryo develops directly from a nucellar or integumental non-reduced cell, while in gametophytic apomixis, the embryo sac is formed from a nucellar non-reduced cell. Gametophytic apomixis is also classified as diplosporous and aposporous depending on whether the embryo sac develops directly from the megaspore mother cell (MMC) or from another nucellar cell, respectively. Apomixis in angiosperms is distributed across 78 families and 293 genera, which represent approximately 2.2% of the total [49].

At least three species of the genus Eragrostis have been described as apomictic: *E. curvula*, *E. heteromera*, and *E. chloromelas* [24]. Other species such as *E. tef*, *E. cilianensis*, and *E. intermedia* are sexual [24,50]. *E. curvula* has a unique diplosporous apomixis called Eragrostis type [51]. The combination of a complete lack of meiosis together with two rounds of mitosis instead of three in the embryo sac development makes this type of apomixis distinctive. Other types of apomixis also include lack of meiosis (Antennaria type) or two rounds of mitosis (Panicum type); nevertheless, the Eragrostis type is the only one that contains both a lack of meiosis and two rounds of mitosis [51]. The final seed embryo:endosperm ploidy ratio in the apomictic Eragrostis type is 2:3, like in sexual seeds. The endosperm is produced by the fusion between a mononucleated non-reduced central cell and a reduced sperm cell, while the embryo is developed by parthenogenesis from the non-reduced egg cell.

Several advances were made in order to unravel the molecular mechanisms underlying apomixis, and some of them could reproduce apomeiosis and parthenogenesis in mutant or edited sexual plants [52,53]. However, the trait cannot yet be transferred to crops, since the genetic tools used in those approaches are inefficient. Transferring apomixis to economically important crops would revolutionize agriculture as we know it [54,55]. The ability to fix heterozygosity would impact seed production in two ways, (i) allowing fixing hybrid vigour for more than one generation in crops that are actually produced by hybridisation, such as corn, and (ii) fixing the hybrid vigour of crops that are not actually produced by hybridisation, such as wheat or rice. Understanding the apomictic mechanism to control seed development would also allow the propagation by seeds of crops that are reproduced vegetatively, such as strawberry and potato. Even more, it would also allow shortening breeding cycles accelerating the generation of new cultivars, and the seeds produced would target the needs of each region.

It is widely accepted that apomixis evolved as a modification of the sexual pathway in which the silence or failure of mechanisms such sa meiosis lead to the rise of apomixis [46,48,56]. However, other polyphenics theories suggest that apomixis and sexuality coexisted anciently, and full sexual eukaryote species lost the genetic or epigenetic mechanism controlling the switch to increase sexuality under biotic or abiotic stresses [57]. Other interpretations propose that apomixis is a deviation of the sexual pathway resulting from asynchronously expressed duplicate genes that control female development [58,59].

Studies on natural populations near the centre of origin in South Africa showed the variation in *E. curvula* in terms of ploidy and reproduction [60]. In this way, all genotypes found in the area had a basic number of 10 chromosomes and were tetraploid, pentaploid, hexaploid, and octaploid. All the accessions collected here were full apomictic or facultative and no sexual genotypes were found. Other research observed the occurrence of sexuality at diploid level [61] and no apomictic diploid has been found so far. Further studies breeding sexual and apomictic accessions generated sexual tetraploid genotypes [26,27,28,62]. More recently, similar types of crossings were made using sexual and facultative tetraploid genotypes as female and male parents, respectively [37]. The cytoembryological analysis in this study demonstrates the occurrence of sexual, facultative, and full apomictic genotypes in offspring. The availability of the whole range of reproductive modes at the tetraploid level in *E. curvula* made the species an ideal model for the study of diplosporous apomixis. The origin of polyploids in this grass seems to be inherited from the hybridisation of two common ancestors, classifying *E. curvula* as an allotetraploid [60,63,64].

It has been a long time since apomixis was described [65]; however, the availability of new technologies in recent decades allowed us to exponentially increase the knowledge about this trait. *E. curvula* was not the exception, and important advances were made using this species as a model to shed light on apomixis regulation. Through this review, we highlight the advances in *E. curvula* and the importance of the species as apomictic model. We also present hypotheses based on the strong evidence collected in recent years. Finally, we summarise how *E. curvula* changed its status from being orphan crop to being well characterised as a collateral result of being an apomictic model.

## 2. Diplosporous Apomixis and Sexuality in *E. curvula*

Gametophytic apomixis in angiosperms is divided into aposporous and diplosporous. In apospory, the non-reduced embryo sacs arise from somatic cells of the ovule (nucellus), while in diplospory, the non-reduced embryo sac arises directly from the MMC [66]. More recent and detailed classification divides gametophytic apomixis into nine types: Allium; Taraxacum; Ixeris; Blumea; Elymus 1, 2, and 3; Antennaria; Hieracium; Eragrostis; and Panicum [51].

**Figure 1:**
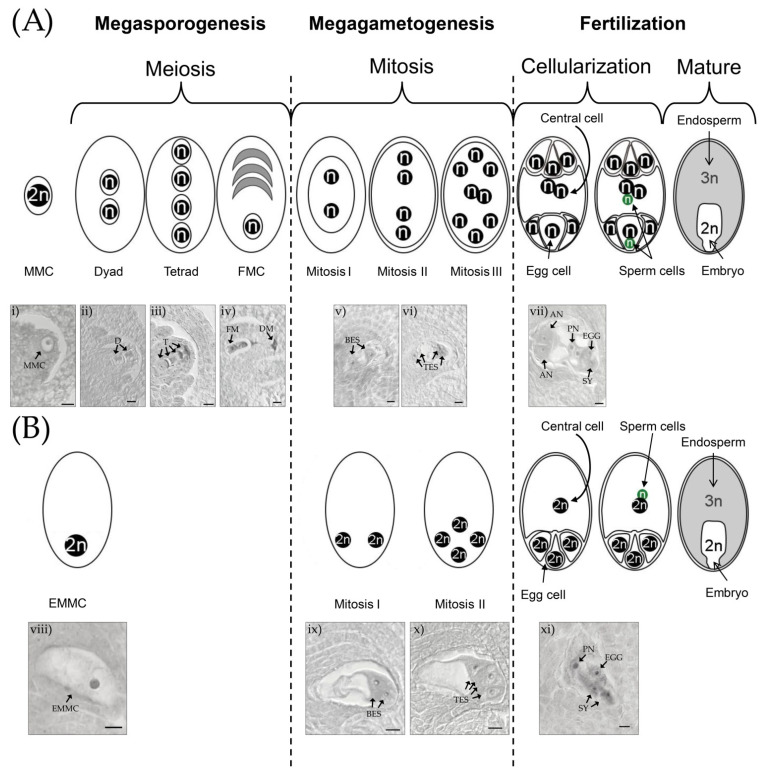
Scheme and safranin-fast green-stained sections of *E. curvula* embryo sac development in (**A**) sexual and (**B**) diplosporous apomictic (Eragrostis type) plants. Note in (**B**) the lack of meiosis during megasporogenesis, the absence of one round of mitosis in megagametogenesis, the fertilization of the polar nucleus (pseudogamy), and the formation of the embryo without fertilization (parthenogenesis). The scale bar equals 10 µm. (**A**) Sexual pathway (i) megaspore mother cell (MMC), (ii) meiosis I showing the dyad (D), (iii) meiosis II with the four cells in a linear tetrad, (iv) functional megaspore (FM) and degenerated megaspores (DM), (v) binucleated embryo sac (BEC) derived from mitosis I, (vi) tetranucleated embryo sac (TEC) from mitosis II, and (vii) embryo sac with antipodal cells (AN), polar nuclei (PN), egg cell (EGG), and one out of two synergid cells (SY). (**B**) Eragrostis type diplosporous apomixis, (viii) elongated megaspore mother cell (EMMC), (ix) binucleated embryo sac (BES) derived from mitosis I with nuclei at the micropylar pole, (x) tetranucleated embryo sac (TES) from mitosis II with nuclei at the micropylar pole, and (xi) embryo sac with polar nucleus (PN), egg cell (EGG), and synergid cells (SY).

The sexual embryo sac development of *E. curvula* is a typical Polygonum type (Figure 1). At the beginning of sporogenesis the archesporal cell differentiates into MMC, which then enter into meiosis I, producing two cells (dyad) and four reduced and recombined cells after meiosis II (tetrad). Three of these cells degenerate, and the functional megaspore gives rise to the future embryo sac. When the gametogenesis starts, the functional megaspore goes through three rounds of mitosis, creating an octonucleated (seven-celled) embryo sac composed of: three antipodal cells, two synergid cells, one central cell (with two polar nuclei), and the egg cell. Finally, once the pollen tube reaches the embryo sac, one sperm cell of the pollen fertilizes the egg cell and the other the central cell, resulting in double fertilization and giving rise to the embryo and endosperm, respectively. The maternal:paternal contribution in this sexual development is 2:1 in the endosperm and 1:1 in the embryo.

In the Eragrostis type of apomixis, meiosis is completely missing (apomeiosis), and the embryo sac develops from the non-reduced MMC, which immediately starts to grow, producing a diplosporous embryo sac (Figure 1). After only two rounds of mitosis, the embryo sac continues enlarging and contains four non-reduced nuclei at the micropylar pole. Then, three nuclei are cellularized, producing the egg cell and two synergid cells. The remaining nucleus is free in the cytoplasm of the central cell. The embryo sac grows to the same size as sexual embryo sacs [67] and develops an embryo without fertilization, by parthenogenesis. The endosperm development does not occur until anthesis, because the polar nucleus needs to be fertilized by a sperm cell (pseudogamy). The maternal:paternal contribution of the endosperm and embryo are 2:1 and 2:0, respectively; as an example, the endosperm of a tetraploid cultivar has 40 maternal plus 20 paternal chromosomes and the embryo has 40 maternal. However, thanks to the two rounds of mitosis, the embryo:endosperm ratio is the same in sexual and apomictic seeds of *E. curvula* (2:3). It is important to note that in *E. curvula*, as well as in the majority of apomictic species, normal reduced microspores are produced by meiosis during the pollen development [63].

## 3. Advantages of the *E. curvula* Apomictic Model

The differences between the Eragrostis type and the other types of embryo sac development reported by Crane [51] make this model very attractive to transfer to major crops. For instance, the complete loss of meiosis in the MMC seems to be less complex in terms of regulation. In other types of apomictic development, such as in Taraxacum, division of the MMC usually starts with a modified meiosis that produces a non-reduced sporocyte [68]. In Panicum, as in other aposporous apomictic species, meiosis takes place normally in the MMC, but one or more embryo sacs develop from the adjacent nucellar cells. In these types of apomixis, in which meiosis is still present at the MMC, the regulation seems to be more complex than in the Eragrostis type, since the non-reduced sporocyte is produced by a disruption of meiosis or is initiated in a cell distinct from the MMC. In the Eragrostis type, the genes responsible for meiosis seem to be silenced in apomictic pistils, since a meiotic process never starts in an apomictic MMC [42,51]. Finding the genes responsible for the lack of meiosis that are repressed or not present at all in *E. curvula* full apomictic plant genomes could simplify the discovery of the operating mechanism and possibly its transference to major crops. Even more, the ability to control meiosis only in the female gametophyte will give more flexibility to breeders, since it could be possible to switch the meiotic process on/off.

As was mentioned before, during apomictic megagametogenesis in *E. curvula*, a four-celled embryo sac is produced after two rounds of mitosis. The central cell developed by this mechanism is not reduced and it has only one nucleus. This means that after fertilization the endosperm will have the same genome ratio in sexual and apomictic seeds in *E. curvula*. In other types of apomixis, where three rounds of mitosis produce a non-reduced octonucleated embryo sac, two non-reduced polar nuclei are fertilized by a reduced sperm cell of the pollen. The embryo:endosperm ratio produced by this process is 2:5, while in the sexual counterpart, the ratio is 2:3. This change in the ratio is due to the increased maternal contribution in the endosperm. This unbalanced ratio has been reported in species of the genera Paspalum, Boechera, and Ranunculus [69,70,71].

The conservation of the genome ratio in the endosperm in sexual and apomictic genotypes in *E. curvula* is a very important aspect to transfer apomixis to major crops. The regulation of gene expression during imprinting (i.e., monoallelic expression of a small subset of genes depending on their parent of origin) is mediated by epigenetic mechanisms, and, in general, one of the alleles is repressed by cytosine methylation [72,73]. The maternal:paternal genome ratio during the imprinting in the endosperm seems to be essential for seed development, while the embryo can be developed without imprinting [74,75]. This is especially important in cereals, which are the main group of target crops to transfer apomixis, constituting 33% of the total food production [76]. Cereals are very sensitive to changes in the maternal and paternal contributions to endosperm. For instance, in *Zea mays*, the maternal:paternal ratio for a normal endosperm must be 2:1. Normal seeds can arise when the ploidy increases to 4:2, showing the importance of the 2:1 relationship [77]. Changes in the relationship of the maternal:paternal ratio produces endosperm failure and embryo abortion [74]. In rice, it was found that maternal and paternal genomes have different functions in the regulation of endosperm development. The maternal genome is involved in endosperm cellularization, whereas the paternal genome delays or inhibits cellularization [78]. In rice, the unbalance in the ploidy ratio of embryo and endosperm produces seed abortion, while polyploid embryo and endosperm with balanced parental genomes are usually viable [79]. The use of *E. curvula* as a model species to study apomixis has remarkable advantages to transfer the trait to major crops such as corn and rice since the relationship between the maternal and paternal genome in the endosperm is maintained.

Another point to keep in mind is that in adventitious apomixis, one or several embryos develop from nucellar or integuments somatic cells in parallel or after sexual embryogenesis, resulting in more than one embryo within a seed [80]. The development of more than one embryo per seed is not desirable to transfer the trait, since, possibly, apomictic and sexual embryos could arise from the same seed. In diplospory, compared to apospory and adventitious embryony, the chances of polyembryony are lower [81,82,83].

## 4. Methods for Assessing the Reproductive Mode. in *E. curvula*

In order to accurately and quickly assess the reproductive mode of *E. curvula* plants, different techniques have been used [36]. Cytological analysis involves microscopic observation of paraffin or resin-embedded, sectioned material, which is usually stained with safranin-fast green to study embryo sac development. In this way it is possible to differentiate the cellular and nuclear organization between sexual and apomictic embryo sac development. This is a quantitative method, since the rate of sexual and apomictic embryo sacs can be calculated. On the other hand, is very laborious and time-consuming and only takes into account apomeiosis. Cytoembryological analyses can also be performed through tissue clarification and differential interference contrast (DIC) microscopy, avoiding the embedding, sectioning, and staining of the spikelet. The progeny test is another useful strategy since it examines the variability or uniformity of descendants of a plant by morphology or molecular markers. The main advantage of the progeny test is that it evaluates apomeiosis and parthenogenesis. Flow cytometry seed screening can analyse a large number of samples in a short time by detecting differences in ploidy between seeds produced by apomictic and sexual processes. In *E. curvula*, this technique did not differentiate between sexual and apomictic plants, since the embryo:endosperm DNA content ratio was similar in both seeds [36]. Previous studies indicated that the presence of the polysaccharide callose on the cell wall of the MMC is associated only with sexual processes [84,85]. Nevertheless, our results showed callose deposition in apomictic genotypes, although the pattern found in apomictic embryo sacs was clearly different from the sexual, allowing discrimination between sexual and apomictic pistils [36]. While many techniques have been shown to be useful to classify *E. curvula’s* reproductive mode, cytoembryological analysis and progeny tests are the most precise and informative techniques because of their capacity to discriminate quantitatively between apomictic and sexual plants. New genetic markers are being developed in order to distinguish between sexual and apomictic genotypes; however, it will be hard to develop a technique to assess the quantitative relationship between apomictic and sexual embryo sacs in facultative genotypes.

## 5. Ploidy

One of the main advantages of using *E. curvula* as a diplosporous apomixis model is the presence of full apomictic, facultative, and sexual genotypes at tetraploid ploidy level (Table 1). The only genotype that was not reported as found in nature is the sexual tetraploid. Nevertheless, by crossing a sexual diploid with an apomictic tetraploid, it was possible to obtain the sexual tetraploid OTA-S, suggesting that this genotype could occur naturally [27]. In 1974, a facultative apomictic accession was found in nature in South Africa [62]. Another cytogenetic study of natural populations of *E. curvula* growing at different altitude levels reported the presence of full apomictic genotypes in the higher and lower altitudes, while in the transition area, both facultative and full apomictic genotypes were found, being all the analysed plants’ polyploids [60]. Polyploids are typically classified as autopolyploids and allopolyploids, depending on the origin of chromosome duplication. If the duplication is derived from a structurally similar, homologous chromosome set of the same species, the polyploid is classified as autopolyploid, whereas if the duplication comes from the hybridisation of non-homologous chromosomes from different species is defined as allopolyploid. In practice, the polyploids are classified by observing the pairing of their chromosomes during pollen meiosis since autopolyploids present polyvalent pairing and in allopolyploids bivalent. The observations of bivalent pairings during meiosis in tetraploid and hexaploid genotypes collected in nature indicate that *E. curvula* is an allotetraploid species [60]. Other analysis performed over 13 accessions of *E. curvula* including tetraploid, hexaploid, heptaploid and octaploid karyotypes showed a high frequency of bivalents [64]. Interestingly, some cultivars showed the absence of polyvalents, and others showed a low frequency, meaning that both allotetraploid and segmental allotetraploid genotypes probably exist. More evidence of the segmental allotetraploidy comes from the Catalina cv., which presents a high proportion of bivalents pairing and a reduced quantity of polyvalents [63].

The first collected *E. curvula* diploid genotype was in Kimberley, South Africa. However, only the chromosome number was recorded and the reproductive mode was not assessed [86]. The second one was collected in 1964 in Middelburg, South Africa, (USDA accession PI 299928), and the reproductive mode was assessed, demonstrating its sexuality [61]. Finally, six diploid genotypes were found in nature in South Africa [87,88]. These findings suggest that the progenitors of the allotetraploids could be still present as in other species of the genus such as *E. tef,* in which one of the progenitors was already identified [11]. However, more detailed studies over natural populations of *E. curvula* need to be carried out to confirm its existence.

All diploid genotypes analysed so far were classified as sexual [62,87,88]. Even more, in vitro culture of immature inflorescences of the apomictic tetraploid Tanganyika INTA cv. led to the regeneration of a sexual diploid plant, Victoria cv. [31]. The mechanisms driving the di-haploidization of Tanganyika are not clear. Cardone et al. [31] suggested three hypotheses: (i) its development from a microspore in the correct stage, (ii) somatic haploidization, and (iii) haploid parthenogenesis (a common mechanism in apomictic species). In general, di-haploidization involves changes in genome organization that eventually creates pairs of homologous chromosomes that are paired with each other, limiting the homoeologous pairing [89]. Tanganyika INTA displays a variable number of polyvalent and bivalent chromosomes pairing [64]. Polyvalent pairing is less stable than bivalent and can lose chromosomes during anaphase [90,91]. In vitro culture could be the cause of the failure of multivalent pairing in the meiosis of Tanganyika INTA that gave rise to the di-haploid plant. The reason why an apomictic diploid did not arise from the in vitro cultured explant could be the absence of the region in the microspore that gave rise to Victoria or by the absence of the epigenetic landscape necessary for the expression of apomixis. Another hypothesis proposes that apomixis is linked to a recessive factor that is masked in polyploids and lethal in diploids [92].

Diploids produce very low amounts of seeds when they are self-pollinated. For instance, the mean seed production for diploids is 0.005 g (g) (min = 0 g, max = 0.050 g) per plant, while in tetraploids it is 0.941 g (min = 0.305 g, max = 1.530 g). Although the comparison between diploids and tetraploids is confounded with grain size, the more than 100-fold difference in seed yield appears large enough to indicate a substantially lower seed set for the diploids [61]. The same behaviour was observed in accessions maintained at CERZOS-CONICET collection by our group in which the number of seeds per season in diploids is remarkably lower than in tetraploids (data not published). Regarding seed weight, the diploid Victoria cv. produces seeds that are lighter and smaller than those produced by plants belonging to the tetraploid Tanganyika INTA cv. (weight of 100 seeds are 23 and 40 mg, respectively) [31]. In this way, we can hypothesize that polyploidization in *E. curvula* could be a “scape” due to failures in normal sexual pathways that we can observe in diploids that show low seed production [61]. Polyploidy might be required in the formation of apomictic species because a diploid or aneuploidy gamete is necessary for the transmission of genes that cause apomixis. Apomictic diploids are rare but were found in nature in a few species. The origin of these genotypes is associated with polyploids and is still in discussion since some works postulate that apomictic diploids arise from hybridisation between polyploid and diploid genotypes, whereas others suggest that they are a consequence of apomeiosis in the female and male gametophyte [93,94].

## 6. Apomixis Inheritance in *E. curvula*

**Figure 2:**
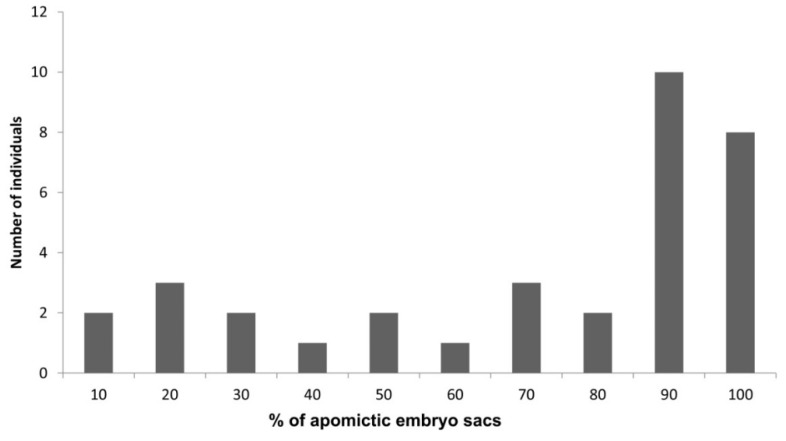
Percentage of apomictic embryo sacs in apomictic F1 hybrids individuals of the mapping population coming from the cross OTA-S (sex) × Don Walter (apo).

Crosses between tetraploid *E. curvula* plants with contrasting reproductive modes were made on different occasions in order to understand the inheritance of apomixis and its genetic control. The first population was developed by crossing sexual (female parent) and apomictic tetraploid (pollen donor) plants [26]. The resulting F1 population showed a segregation ratio apomictic:sexual of 1:1. However, this result was recently corrected to 1.7:1 (apo:sex) since the presence of at least one apomictic embryo implies that the plant is apomictic, and in this work [26] the genotypes were initially divided into apomictic, highly sexual, and sexual instead of apomictic and sexual. Another tetraploid F1 population was derived from the crosses between a natural apomictic genotype and a synthetic sexual tetraploid plant obtained from a colchicine treatment of a natural sexual diploid [63]. Interestingly, the 85 F1 plants resulting from this cross were sexual. If genetic recombination controls the apomictic:sexual ratio, then in this case it favours the sexuality over apomixis. The factors that favour the sexuality in this case are not clear. One point to take into account in this work is that the mother plant could not produce seeds in isolation or self-pollination; however, the hybrid nature of the offspring was not analysed. Our evidence indicates that the mentor effect occurs in *E. curvula,* in which the presence of external pollen favours self-pollination [95]. A new population was developed by our group at CERZOS-CONICET by crossing the sexual tetraploid OTA-S plant with the facultative apomictic tetraploid Don Walter cv. [37] (Figure 2). Different cultivars were tested to be used as pollen donors, with Don Walter showing the highest seed production while some cultivars such as the full apomictic Tanganyika USDA were non-compatible with the sexual plant. The offspring of this F1 population was tetraploid as well as the parents and the ratio between apomictic and sexual plants was 1:1, and the percentage of diplosporous embryo sacs in the progeny plants ranged from 3% (facultative) to 100% (full apomictic) (Figure 2).

This population is a very useful tool since the availability of sexual tetraploids before this work was bounded. On the other hand, we have now a group of closely related plants with different reproductive modes, which are perfect to perform comparative analyses in terms of morphology, cytology, genetics, transcriptomics, genomics, and epigenetics analyses.

## 7. The Diplospory Genomic Region

Studies of different apomict species such as *Pennisetum squamulatum* [96], *Taraxacum officinale* [68], and Paspalum [97] agree that apomixis is dominant over sexuality and that the genes governing the trait co-segregate in a single region. The same hypothesis has been proposed in *E. curvula,* and some advances were made in the identification of the genomic region.

In order to identify and characterise the genomic region associated with apomixis in *E. curvula,* a linkage map coming from the cross OTA-S x Don Walter was constructed [37]. The offspring of this cross rendered 62 hybrids, which were genotyped by sequencing (GBS). The phenotypic characterisation of this population was made by cytoembryology. Using this data linkage, maps for Don Walter and OTA-S were constructed, resulting in 40 linkage groups each. Interestingly, four GBS-SNPs markers on linkage group 3 of Don Walter co-segregate with apomeiosis, indicating that it is controlled by a single locus but, we do not have evidence of whether parthenogenesis is controlled by the same region or an additional region is involved but not yet detected [37]. Near this region, two QTLs were mapped that could regulate some quantitative aspects of the apomictic reproduction.

**Figure 3:**
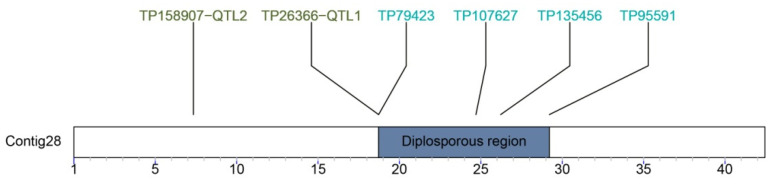
Region linked to apomixis in *E. curvula* (Victoria cv.) with four linked GBS-SNPs markers (blue label) and two mapped QTLs (green label).

In parallel to the construction of this first linkage map for the species, the genome assembly of the diploid sexual genotype Victoria was performed [39]. This genome assembly was the starting point to sequence more complex apomictic tetraploids. The sequencing of the genome was generated through a combination of PacBio, Chicago, and Hi-C DNA libraries, which rendered a high-quality chromosome-scale assembly with an N50 of 43 Mb. Even though the Victoria cv. is sexual, it was produced from inflorescences in vitro culture of a Tanganyika INTA plant (facultative apomictic); hence, the genomic background should be similar. The four markers linked to apomixis and the two QTLs were aligned to a single contig in the Victoria genome assembly (Contig28), which in turn represents a whole single chromosome (Figure 3).

The region defined by the markers has 10,492,413 bp in the Victoria genome assembly. In *Pennisetum squamulatum*, the region associated with apomixis comprises a quarter of a chromosome with a length of 50 Mb [98]. A syntenic analysis is performed between the region associated with apomixis in Victoria and in the tetraploid apomictic genomes assemblies Don Walter and Tanganyika INTA cvs. (data not published). Even when the apomictic assemblies are more fragmented than Victoria, it was possible to detect in several contigs some syntenic fragments to the region in Victoria and some fragments that are specific to the apomictic genotypes. Three possible scenarios could explain the presence of the markers linked to apomixis in the diploid sexual Victoria: (i) the region is present in one of the four homeologous chromosomes of the tetraploid apomictic genotypes of *E. curvula,* but the markers are present in the all of the other three as well. (ii) Some parts of the apomictic region are present in Victoria, but the complete region is only observed in polyploids. (iii) The genomic region is similar in apomictic and sexual genotypes; however, the epigenetic landscape is different. The best characterised region associated with apomixis was made on the aposporous grass species *Pennisetum squamulatum*, being hemizygous and covers a substantial part of a chromosome [99]. Even though non-recombination was found in this region it exhibits multiple regions of micro-synteny with *Sorghum bicolor* and *Oryza sativa* [100]. Similar results were found in *Paspalum simplex* since two contigs associated with the apospory region showed micro-synteny with *Setaria italica* and *Sorghum bicolor* [101]. This syntenic behaviour is similar to that observed in *E. curvula* apomictic and sexual genomes, in which regions of micro-synteny were found together with regions that are specific to apomictic genotypes.

## 8. Plasticity in the Apomictic: Sexual Frequency

Apomixis usually occurs in a facultative form; that is, apomictic individuals produce sexual and apomictic seeds at the same time. In natural and synthetic populations, the majority of the genotypes are facultative [26,36,37,63]. This behaviour seems to be common in the majority of the apomictic species, and the frequency of the ratio between apomictic and sexual embryo sacs is not constant over the different flowering seasons [57,71,102,103,104,105,106]. In *E. curvula*, abiotic stress conditions can alter the frequency of apomictic:sexual ratio in facultative cultivars, increasing the percentage of sexual embryo sacs, even if not all of the sexual embryo sacs lead to viable seeds [42]. This response could be found not only in plants, but in several kingdoms, and it is reasonable to expect that facultative apomicts tend to switch to sexual reproduction more often under stress conditions and that such a stress-dependent switch facilitates the organism’s adaptation to a stressful environment [57].

## 9. Gene Expression in Apomictic and Sexual Genotypes

The first approach to assess the overall gene expression profile in *E. curvula* was made using expressed sequence tags (EST) [33,34]. Inflorescences from Tanganyika INTA cv. (apomictic tetraploid), Bahiense cv. (sexual tetraploid) and Victoria cv. (sexual diploid) and leaves from Tanganyika were sequenced and compared. The sequencing output was 12,295 ESTs corresponding to 8884 unigenes. This work allowed the discovery of *E. curvula* genes for the first time and to analyse the differential expression between plants with different reproductive modes and ploidy levels. In this way, eleven genes were found differentially expressed between genotypes with different reproductive modes, including methyltransferases, cysteine synthase, ribosomal protein L2, rubisco large subunit within others. In addition, 254 SSR and 190 SNP markers were developed from the ESTs.

The development of NGS technologies allowed us to analyse the whole expression profile of inflorescences with contrasting reproductive mode, Tanganyika USDA (full apomictic) and OTA-S (sexual), giving rise to the first reference transcriptome from flowers of the species [35]. The transcriptome obtained through the Roche 454 technology yielded 49,568 transcripts, of which 6203 were over-expressed for apomixis and 3060 were down-regulated. The differentially expressed genes in this work where clustered in GO terms and were functionally annotated. In this work, 11,475 SSRs were also identified and several of them were validated in *E. curvula* germoplasm using SSR-based primer. This transcriptome is a very useful tool for mining genes, design primers, and discover candidates related to the reproductive mode.

The rate of apomictic and sexual embryo sacs under different stress conditions was assessed showing an increase in sexual embryo sacs under different stress situations [42]. In order to assess the relationship between gene expression and the increase in sexual embryo sacs under stress, inflorescence transcriptomes of plants exposed to drought stress were sequenced using Illumina Hi-Seq technology [45]. The assembly outcome consisted of 201,011 transcripts, out of which 350 were down-regulated and 151 were up-regulated in the stressed plants. Among the up-regulated genes, five were found to be related to the ubiquitin pathway. Another interesting gene over-expressed under stress conditions that will be discussed in the epigenetic section was ROS1, a de-methylating gene [107]. Six of the down-regulated genes were annotated as F-Box proteins, which are also involved in the ubiquitin degradation pathway. This work gave us an overview of the pathways involved in the quantitative regulation of the apomictic:sexual ratio observed in facultative cultivars. Even more, the number of unique genes expressed in the Don Walter transcriptome complements the previously reported reference transcriptome [35].

Other transcriptomes of the species had been sequenced, such as 17 accessions from leaves sequenced in order to identify genes related to the resistance of *E. curvula* to *Magnaporthe oryzae* (N. Talbot and M Moscou, Sainsbury Laboratory, the UK, as was reported by Carballo et al. [108]).

## 10. Epigenetics Regulation of Apomixis

Epigenetics is defined as the heritable changes in gene expression that are not coded in the DNA sequence [109]. However, modern definitions include long-term alterations in the transcriptional potential of a cell that are not necessarily heritable. The main mechanisms associated with epigenetics are histone modifications, DNA methylation and RNA interference [110]. The mechanism associated with the specification and development of germline in plants has not been fully elucidated so far, even though great advances were made to understand these mechanisms. However, epigenetic mechanisms such as DNA methylation, histone modifications, and small RNAs were involved in plant silencing germline genes. Even more, some of these genes were found differentially expressed between male and female germlines [111,112]. Several authors assume that apomixis emerges as a consequence of the repression of sexual processes [46,48,56,58], and epigenetics was pointed out as the cause of this repression since the process could be reversible, modulating the percentage of apomictic embryo sacs under certain conditions.

A few decades ago, apomixis was studied, mainly taking into account the genetic determination of the trait [59,113]. However, more recently, apomixis was approached from the epigenetic side since the trait was found to be more complex than was originally thought [114,115,116]. Several studies in *E. curvula* introduced new evidence of the epigenetic nature of some aspects of this particular reproductive mode. The first line of evidence showed that the number of methylated sites was higher in tetraploid genotypes than in diploids [40]. Further analyses comparing natural and synthetic tetraploid genotypes, showed higher levels of methylation in colchiploid tetraploids, i.e., tetraploids obtained from diploids using colchicine [41].

Moreover, it is widely known that epigenetic regulation takes place under different biotic and abiotic conditions [117], and it was shown that in facultative *E. curvula* plants, stress triggers the increased sexuality [41,42,45], as in other apomictic species [57,71,102,103,104,105,106]. This mechanism is used as a “scape” of clonal reproduction to increase the genetic variability and the chances of surviving in hostile environments [49]. In *E. curvula* it was shown that the frequency of sexual ovules increases under different abiotic stresses. In facultative apomictic Tanganyika INTA plants growing under drought stress conditions, the percentage of sexual embryo sacs increased from 2.4% to 14.4% [42]. Similar results were observed in plants of Tanganyika INTA cv. regenerated in vitro. in which the frequency of sexual reproduction increased to 33.3% compared with the plants that produced the seeds used for the in vitro culture. In full apomictic genotypes, sexual embryo sacs were not observed, neither in plants obtained by in vitro culture nor in plants under water stress conditions [42]. In vitro culture treatments are also considered a source of stress [118]. Another interesting observation was that when the normal conditions are restored after the stress, the percentage of sexual embryo sacs starts to decrease, reaching after some time the normal values for the genotype [42]. This phenomenon was also observed in plants where the chromosomes were doubled by colchicine, which is also considered a genomic stress [41]. The first generations of these plants were variable, and the plants were classified as sexual, but then, the number of apomictic embryo sacs started to increase, decreasing the percentage of sexuality in four years to 20%. Methylation-sensitive amplification polymorphism profiles between panicles of drought and normal irrigated plants showed that the increase in sexual embryo sacs positively correlated with global methylation changes [42]. This can be explained by a plastic mechanism, such as DNA methylations and de-methylations, as was mentioned before.

**Figure 4:**
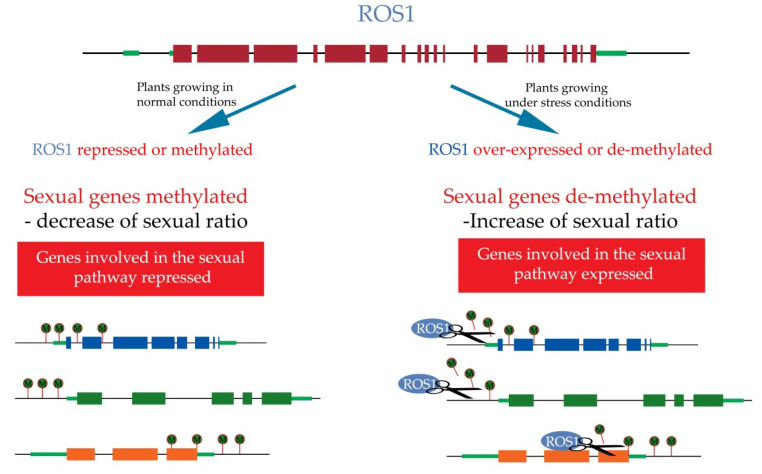
Hypothetical model of the quantitative regulation of diplosporous apomixis mediated by ROS1. Green circles attached to the genes represent methylated positions over putative genes involved in the sexual pathway. This situation is observed when plants grow under normal conditions and ROS1 is repressed or de-methylated. Blue ovals represent ROS1 de-methylating genes involved in the sexual pathway when plants are exposed to stressful conditions. As a consequence, the sexual ratio increases.

Transcriptome profiles of drought-stressed and normal watered plants of the facultative Don Walter cv. were also compared. Interestingly, the gene ROS1, involved in de-methylation [107] was found to be over-expressed in stressed plants [45]. This means that genes usually methylated in normal conditions are being de-methylated under stress by ROS1. We suggested that ROS1 regulates genes involved in sexual pathways that are methylated in apomictic genotypes, i.e., silenced in normal conditions, and de-methylated under stress, allowing the increase in sexual processes (Figure 4).

ARGONAUTEs (AGOs) are effector proteins involved in the small-RNA pathways, variable in number depending on the species [119]. AGOs are actively involved in silencing processes since small RNAs are loaded onto AGO proteins and also participate in the methylation pathway, recruiting *DRM2* to start the methylation process [120,121]. In *E. curvula,* it was found that *EcAGO104* has different expression patterns between full apomictic and sexual plants [43]. In archesporal stages, the expression did not show variation between both genotypes, whereas in gametophytic stages the expression in the apomictic genotype was higher. In situ hybridisation also showed different spatial expression patterns in which *EcAGO104* is expressed from the archesporal cell to mature embryo sacs in apomictic plants, whereas in sexual ones, its expression starts from the functional megaspore. It was postulated that the expression of *EcAGO104* during sporogenesis prevents meiosis and promotes gametophytic development [43], as was observed in other species [122].

*EcDMT102* and *EcCHR106* genes, involved in methylation, were found differentially expressed in archesporal and gametophytic stages between sexual and apomictic genotypes [43]. *EcDMT102* was found to be more expressed in sexual genotypes while *EcCHR106* was found in apomictic ones. *DMT102*, which is also called *ZMET2* in *Z. mays*, is responsible for the maintenance of methylation in CHG contexts [123], and the orthologous gene of *CHR106*, *DDM1* from *A. thaliana*, allows DNA methyltransferases to access H1-containing heterochromatin in *A. thaliana* [124]. Even when the overall methylation level in the sexual genotype is lower than in the apomictic ones [46], some genes such as *DMT102* that are responsible for the maintenance of methylation are more expressed in sexual genotypes. A plausible explanation could be that *DMT102* silences other genes involved in sexuality repression. However, more evidence is needed in order to support this hypothesis.

The whole small-RNA profiles of a full apomictic and a sexual genotype were also analysed in *E. curvula* [44]. In order to detect small RNAs associated with the reproductive mode, differential expression analysis was performed using the same RNA that was used to sequence the reference transcriptome [35], where the small-RNA targets were mined. Three known miRNA were identified, mir821, mir5049, and mir8175 targeting a MADS-box transcription factor 6, an unknown transcript, and a transposable element, respectively. Interestingly, mir821, which is overrepresented in the sexual genotype, targets a MADS-box that was amplified only in the apomictic genotype. MADS-box transcription factor 6 was already described in rice as being involved in meristem cell fate determination and as a regulator of floral organ identity [125].

The whole methylation profile was assessed in inflorescences of *E. curvula* of full apomictic, facultative and sexual tetraploid genotypes using MCSeEd (Methylation Context-Sensitive Enzyme ddRad) technique [46]. The differentially methylated positions between full apomictic vs. sexual, facultative apomictic vs. sexual, and full apomictic vs. facultative genotypes were evaluated in CG, CHG, CHH, and A contexts. One of the main findings in this work was the presence of differentially methylated regions in *ROS1* gene in apomictic vs. sexual comparisons. *ROS1* is responsible for the de-methylation in cytosine’s contexts, usually de-repressing gene expression [107]. This gene was found to be more methylated in apomictic than in sexual genotypes in adenine contexts. Even though the correlation of adenine methylation and gene expression is not fully clear, it was found that gene expression correlates negatively when genes are methylated in the promoter region and positively when they are methylated in the gene body [126]. If this is also true in *E. curvula*, the silencing of *ROS1* would affect the expression of genes involved in the sexual pathway, being silenced in apomictic genotypes. Under stress conditions, *ROS1* is over-expressed, probably inducing the de-repression of sexual pathways, providing evidence of the existence of both functional pathways in facultative apomicts [45]. In this way, we can hypothesize that *ROS1* functions as a quantitative regulator of apomictic:sexual embryo sacs ratio through the de-repression of genes involved in sexual pathways (Figure 4). Other genes associated with apomixis in previous studies, such as those involved in ubiquitin [127,128] and auxin regulation pathways [45,129], were also found differentially methylated between apomictic and sexual genotypes.

## 11. State of the Art of Diplosporous Apomixis in *E. curvula*

In the last few years, advances were made in terms of genetics and epigenetics mechanisms involved in apomixis regulation in *E. curvula*. The emergence of new genomic technologies allowed the identification of genes, regions, and pathways that could be governing apomixis in this grass. The knowledge gathered from the studies in *E. curvula* agrees with findings in other apomictic species, but it was possible to find characteristics that are specific to *E. curvula* or that could be present in other apomictic species and were not yet found.

The main findings in this grass were:

(i) Diplosporous apomixis is governed by a genomic region covering an area of 10.5 Mb that co-segregates with four molecular markers. This region seems to be non-recombinant and some of the genes located there are present exclusively in the apomictic genome of this grass and were not found in any *E. curvula* sexual accession.

(ii) Methylation and small RNAs mechanisms are involved in the regulation of apomixis, being sexual, facultative, and full apomictic genotypes that are differentially methylated. *ROS1* seems to have a major role in the regulation by methylation and de-methylation of key genes related to the apomictic:sexual switch in facultative genotypes. Different miRNAs were found silencing genes in the full apomictic genotype and probably involved in sexual pathways.

(iii) Methylation levels were higher in apomictic genotypes than in sexual, and facultatives were more methylated than the full apomictic ones.

(iv) Differentially expressed genes between sexual and apomictic genotypes and between diploid and tetraploid genotypes were found. The identified candidate genes are functionally characterised. Changes in temporal and spatial gene expression were also observed.

(v) Different types of internal and external stresses change the apomictic:sexual ratio in facultative apomict, increasing the percentage of sexual embryo sacs. This was related to methylation changes occurring as a consequence of stress.

## 12. Conclusions

Here, we summarised the advances made to unravel the control of apomixis using *E. curvula* as a model species for diplospory due to the the peculiarities of the Eragrostis-type embryo sac development. In the last few years the following tools were developed:

- Forty-two transcriptomes from leaves and inflorescences;
- Four small-RNAs libraries;
- The first linkage map for the species, highly saturated, with GBS-SNPs;
- Three genome assemblies, one diploid and two tetraploids;
- More than 4500 SSRs;
- The whole methylation profiles of full apomictic, facultative, and sexual genotypes;
- One genomic region and candidate genes for apomixis.

These tools have changed the status of *E. curvula* from orphan to model species for diplosporous apomixis.

Until now, the main bulk of evidence points out that apomixis is inherited as a single Mendelian factor and is regulated by a few genes in a region. However, these genes seem to be controlled by complex mechanisms in a cascade of reactions. The work in progress aims at finding if the genes present in the region of apomictic genotypes are directly or indirectly involved in the rise of the apomictic components. Some of the candidate genes found in the diplosporous apomictic region of *E. curvula* were not yet described. The functional characterisation of these genes is being conducted, and this information will provide further evidence of the mechanism controlling diplosporous apomixis.

## Figures and Tables

**Table 1:** *E. curvula’s* accessions available at CERZOS-CONICET in which different studies were performed. SSRs: single sequence repeats markers. GBS: genotyping by sequencing. ESTs: expressed sequenced tags. MCSeEd: Methylation Context Sensitive Enzyme ddRad. DArT-seq: Diversity array technology sequencing. * Unpublished.

Accession	Ploidy	Reproductive Mode	Molecular Resources
Victoria	2×	Sexual	Genome [39]/ESTs [33]/SSRs [35,39]
PI299920	2×	Sexual	SSRs [35,39]
PI208214	2×	Sexual	SSRs [35,39]
PI219919	2×	Sexual	SSRs [35,39]
PI219928	2×	Sexual	SSRs [35,39]
Tanganyika USDA	4×	Full apomictic	RNA-seq [35]/SSRs [35,39]/MCSeEd [46]
Tanganyika INTA	4×	Facultative apomictic	ESTs [33]/Genome */SSRs [35,39]
Ermelo	4×	Facultative apomictic	SSRs [35,39]
Morpa	4×	Facultative apomictic	SSRs [35,39]
OTA-S	4×	Sexual	RNA-seq [35]/SSRs [35,39]/GBS [37]/MCSeEd [46]
Catalina	4×	Facultative apomictic	-
Don Walter	4×	Facultative apomictic	RNA-seq [45]/SSRs [35,39]/GBS [37]/Genome */MCSeEd [46]
Bahiense	4×	Facultative apomictic	ESTs [33]/SSRs [33]
62 Hybrids (F1)	4×	Apomictic:sexual (1:1)	GBS [37]/DArT-seq */AFLPs [37]/SSRs [37]
Don Eduardo	6×	Apomictic	SSRs [35,39]
Don Luis	6×	Apomictic	SSRs [35,39]
Kromdraai	6×	Facultative apomictic	SSRs [35,39]
Don Pablo	7×	Apomictic	SSRs [35,39]
Don Juan	8×	Apomictic	SSRs [35,39]
